# Processing speed enhances model-based over model-free reinforcement learning in the presence of high working memory functioning

**DOI:** 10.3389/fpsyg.2014.01450

**Published:** 2014-12-17

**Authors:** Daniel J. Schad, Elisabeth Jünger, Miriam Sebold, Maria Garbusow, Nadine Bernhardt, Amir-Homayoun Javadi, Ulrich S. Zimmermann, Michael N. Smolka, Andreas Heinz, Michael A. Rapp, Quentin J. M. Huys

**Affiliations:** ^1^Department of Psychiatry and Psychotherapy, Charité Universitätsmedizin BerlinBerlin, Germany; ^2^Department of Psychiatry and Psychotherapy, University Hospital Carl Gustav Carus, Technische Universität DresdenDresden, Germany; ^3^Department of Psychiatry and Neuroimaging Center, Technische Universität DresdenDresden, Germany; ^4^Institute of Behavioural Neuroscience, University College LondonLondon, UK; ^5^Area of Excellence Cognitive Sciences, University of PotsdamPotsdam, Germany; ^6^Translational Neuromodeling Unit, Institute of Biomedical Engineering, University of Zurich and Swiss Federal Institute of TechnologyZurich, Switzerland; ^7^Department of Psychiatry, Psychosomatics, and Psychotherapy, Hospital of Psychiatry, University of ZurichZurich, Switzerland

**Keywords:** decision-making, reward, cognitive abilities, model-based and model-free learning, fluid intelligence, habitual and goal-directed system

## Abstract

Theories of decision-making and its neural substrates have long assumed the existence of two distinct and competing valuation systems, variously described as goal-directed vs. habitual, or, more recently and based on statistical arguments, as model-free vs. model-based reinforcement-learning. Though both have been shown to control choices, the cognitive abilities associated with these systems are under ongoing investigation. Here we examine the link to cognitive abilities, and find that individual differences in processing speed covary with a shift from model-free to model-based choice control in the presence of above-average working memory function. This suggests shared cognitive and neural processes; provides a bridge between literatures on intelligence and valuation; and may guide the development of process models of different valuation components. Furthermore, it provides a rationale for individual differences in the tendency to deploy valuation systems, which may be important for understanding the manifold neuropsychiatric diseases associated with malfunctions of valuation.

## Introduction

Habitual responding to rewards and the pursuit of strategic goals both play critical roles in complex human decision-making. Evidence from animal models and human subjects suggests a clear distinction between these systems of behavioral choice. The system of habitual control is reflexive and works on the principles of reinforcement. The goal-directed system, to the contrary, is reflective, and works on the principles of planning. These systems differ in their neural substrates (Killcross and Coutureau, 2003; Yin et al., 2004, 2005) and in their computational characteristics, with goal-directed and habitual systems having features of model-based and model-free reinforcement learning, respectively (Daw et al., 2005; for reviews, see Rangel et al., 2008; Redish et al., 2008; Dolan and Dayan, 2013; Huys et al., 2014). Recent empirical evidence provides initial support for this association (Friedel et al., 2014). Computational accounts (Daw et al., 2005; Johnson and Redish, 2007; Keramati et al., 2011; Huys et al., 2012; Dolan and Dayan, 2013) propose that the model-based system constructs and searches a tree of possible future states and outcomes to compute action values on the fly. Thus, constructing, updating, and searching a decision tree demands fast and flexible information storage, selection of adequate computations, and depends heavily on fast computing power (O'Keefe and Nadel, 1978; Redish, 1999). These components match onto aspects of intelligence including measures of processing speed, working memory capacity, executive control processes and verbal knowledge (Horn and Cattell, 1966; Johnson and Bouchard, 2005; Sternberg, 2012). Yet, how individual differences in specific aspects of intelligence influence the model-based system and its relative dominance over model-free choice remains unclear.

Experimental manipulations involving dual tasks and stress have argued for an important contribution of working memory, with increases in working memory load resulting in a shift away from model-based toward model-free decision-making (Schwabe and Wolf, 2009; Otto et al., 2013a,b), and increases in working memory resulting in a shift toward model-based decision-making (Kurth-Nelson et al., 2012; Bickel et al., 2014). Indeed, both dopamine, which is associated with increased processing speed and working memory function (De Wit et al., 2011; Wunderlich et al., 2012), and the lateral prefrontal cortex (Smittenaar et al., 2013; Lee et al., 2014) appear to be directly involved in model-based choice and affect the relative balance between the systems. Accordingly, recent computational models have proposed important roles for speed/accuracy tradeoffs (Keramati et al., 2011) in the arbitration. We have recently found that individual differences in measures of processing speed and working memory are related to neurobiological markers of model-free learning (Schlagenhauf et al., 2013), and evidence suggests a general involvement of cognitive abilities in choice (Burks et al., 2009). Other accounts of the model-based system suggest a role of verbal task coding (Waltz et al., 2007; FitzGerald et al., 2010), which relates to verbal knowledge (Lehrl, 2005).

Two broad conceptualizations of the structure of intelligence are particularly prominent in the literature. The two-component model (Horn and Cattell, 1966; Sternberg, 2012) identifies two factors that show differential trajectories in developmental studies across the life-span: fluid intelligence, measured by tasks assessing processing speed, working memory, and executive functions; and crystallized intelligence, measured by tests of verbal knowledge putatively acquired through learning from experiences. Comparing children and old adults, measures of verbal knowledge tend to share less variance with abilities such as processing speed and working memory, suggesting that the (culture-based) acquisition of knowledge in children and adolescents as well as the (biologically-driven) decline in processing speed and working memory with age reflect two distinct components of intelligence (Li et al., 2004). The verbal-perceptual-image model (Johnson and Bouchard, 2005) in contrast posits the existence of one general factor together with three subcomponents consisting of verbal knowledge, perceptual speed, and visuospatial rotation abilities. Unlike the two-component model, this conclusion is derived from cross-sectional studies in middle-aged subjects.

Thus, when considering the relationship between cognitive abilities, intelligence, and aspects of decision making, multiple aspects of intelligence need to be taken into account. Importantly, however, both models of intelligence rely on the same key index measures of verbal knowledge, perceptual speed, visuospatial working memory and executive control: the first three reflect principal components of the verbal-perceptual-image model (Johnson and Bouchard, 2005), while the first vs. the last three reflect the crystallized vs. fluid distinction of the two-component model (Horn and Cattell, 1966).

The computational as well as the experimental considerations outlined above suggest that the processes underlying these key index measures may be involved in the model-based system and in its relative dominance over model-free behavior. However, the relative contribution and importance of these different aspects of intelligence is as yet unclear. We therefore examined how the four key cognitive abilities are related to model-free and model-based choice components. We use a two-step Markov decision task that was explicitly designed based on the statistical characteristics of model-based and model-free choice behavior (Daw et al., 2011).

## Materials and methods

### Participants

Twenty-nine adults (8 female; mean age 43.3, range 25–58) participated in our study. Participants were recruited via advertisements in local newspapers and social clubs. Exclusion criteria were any lifetime psychiatric disorder as well as any current medication that could affect cognitive abilities. Demographic information and smoking behavior were recorded. Two subjects were excluded from analyses due to incomplete data. The study was approved by the local ethics committee of the Charité University Medicine Berlin.

### Cognitive ability measures

#### DSST

For the Digit Symbol Substitution Test (DSST), subjects were presented with a code table assigning 9 different abstract symbols to the digits 1–9. Subjects were then presented with a table presenting a list of digits in each top and empty boxes in each bottom row, and instructed to sequentially draw as many of the corresponding symbols underneath the digits as possible in 120 s. DSST scores reflect the number of correct symbols the participants drew within that time. The DSST measures general, unspecific processing speed (Salthouse, 1992), and, to a lesser extent, writing speed, and short-term-memory (Laux and Lane, 1985). In factorial analyses, it has consistently been closely linked to other measures of processing speed such as the Trailmaking Test, part A (Laux and Lane, 1985).

#### Digit span

For the Digit Span test from the Wechsler Adult Intelligence Scale (WAIS-II; Wechsler, 1997), the experimenter reads out increasingly long sequences of digits. Participants were instructed to repeat each sequence in reverse order. The experimenter started with sequences of two digits and increased the number of digits by one until the participant consecutively failed two trials of the same digit span length. The individual Digit Span score represents the number of correctly repeated digits in reverse order. The Digit Span Backwards Task measures verbal working memory capacity in terms of the number of digits a person can memorize concurrently, and the ability to manipulate these items and sequence them in reverse order. Of note, it has been suggested that this aspect of manipulating the order of the digits within working memory has been related to both working memory capacity and visuospatial working memory (Li and Lewandowsky, 1995).

#### TMT

The Trail Making Test (Army Individual Test Battery, 1944) consists of two parts (A and B). In part A, subjects were presented with 25 numbers written in circles distributed across a sheet of paper. Subjects were instructed to use a pencil to connect these numbers in ascending order. In part B, the subjects were presented with both numbers (1–13) and letters (A–L) written in circles distributed across a sheet of paper. As in Part A, subjects were instructed to draw lines to connect the circles in an ascending pattern, but with the added task of alternating between the numbers and letters (i.e., 1-A-2-B-3-C, etc.). Individual TMT A and B scores are the number of seconds required to complete the task. For part B, we computed a ratio score dividing the time needed for completion of part B by the time needed for completion of part A (Corrigan and Hinkeldey, 1987). The Trail Making Test, part A measures visual attention and processing speed, and to a lesser extent, writing speed (Sánchez-Cubillo et al., 2009). Part B, especially the ratio score, has been consistently linked to measures of executive function and task set-switching (Arbuthnott and Frank, 2000) and hence has been shown to be an index measure of executive functions independent of processing speed (Corrigan and Hinkeldey, 1987; Sánchez-Cubillo et al., 2009).

#### MWT

The German vocabulary test (MWT; Lehrl, 2005) consists of 37 lists of 5 verbal items each, of which four represent non-sense words and one represents a correct word. Participants were instructed to mark the correct word in each row without time constraint. Individual MWT scores represent the number of correctly recognized words. Tests of verbal knowledge have been related to education and knowledge domains in healthy and clinical populations (Rolfhus and Ackerman, 1999; Reichenberg et al., 2006) and have been shown to be relatively independent of processing speed and working memory components across the lifespan (Rolfhus and Ackerman, 1999; Li et al., 2004).

### Two-step task

**Figure 1:**
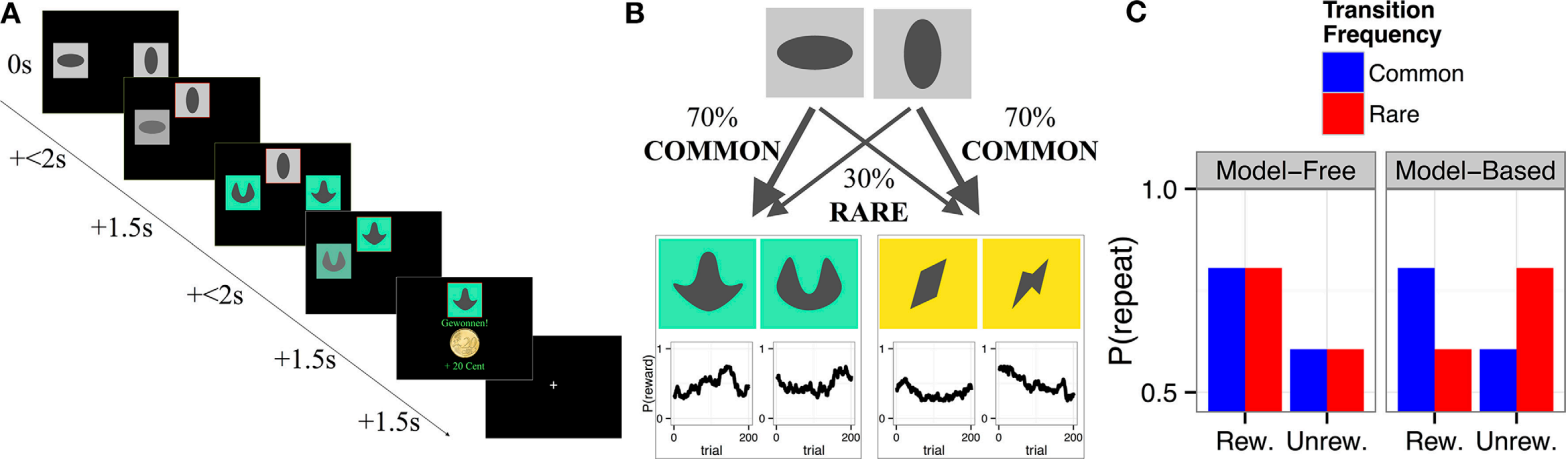
**(A)** Trial structure: Step 1 consisted of a choice between two abstract gray stimuli. The unchosen stimulus faded away while the chosen stimulus was highlighted with a red frame and moved to the top of the screen, where it remained visible for 1.5 s. In Step 2 a second, colored, stimulus pair appeared. Step 2 choices resulted either in a win of 20 Cents or no win. **(B)** Transition structure: Each first stage stimulus led to one, fixed, second stage pair in 70% of the trials (*common transition*), and to the other second stage stimulus pair in 30% of the trials (*rare transition*). Reinforcement probabilities for each second stage stimulus changed slowly and independently between 25% and 75% according to Gaussian random walks with reflecting boundaries (Daw et al., 2011). Win probabilities, P (reward), are displayed as a function of trial number. **(C)** Model predictions: Predictions from the computational model (Daw et al., 2011) based on the model-free (left panel) vs. model-based (right panel) system for the probability to repeat the choice from the previous trial as a function of reward (rew., rewarded; unrew., unrewarded) and transition type at the previous trial. Model-free choice predicts a main effect of *reward*, and no effect of *transition*. Model-based choice predicts an interaction of *transition × reward*. Figure partly adapted from Sebold et al. (2014).

The two-step decision task (Daw et al., 2011; see Figure 1) was re-programmed in MATLAB, using the Psychophysics Toolbox extensions and a different set of colored stimuli. Importantly, the same sequence of outcome probabilities as used in the original publication was used. The task required subjects to choose one of two stimuli (step 1) immediately followed by another stimulus pair at step 2 (see Figure 1A). Participants were instructed to maximize their rewards. Crucially, the probability of reward at step 2 changed over time according to an independent random walk for each of the four step 2 stimuli (Figure 1B). The probabilities of being presented with a given set of stimuli at step 2 were determined by the choice at step 1 and did not change over time; there was a common (70%) and a rare (30%) transition. To enhance participants' motivation one third of all rewards with a fixed minimum of 3 and a maximum of 10 Euros were additionally paid out at the end of the experiment. Participants were given very detailed information about the structure of the task; they were informed about the varying outcome probabilities at step 2 (including being shown sample random walks) and about the constant transition probabilities between step 1 and 2. Subjects underwent 50 practice trials prior to performing the task proper.

### Procedure

Prior to the experiment, participants were screened by telephone. The laboratory test session lasted 1 h in total and started with verbal and written informed consent. After that, participants completed the two-step task, followed by a debriefing questionnaire asking participants for specific strategies, their motivation, and alertness throughout the experiment. Then, participants underwent the neuropsychological testing containing the cognitive ability measures. After completing the Digit Span Backwards Task and the Trail Making Test, participants completed the DSST followed by the German vocabulary test MWT. Finally participants were debriefed and paid out the monetary compensation.

### Analyses

Given an expected true correlation between cognitive ability scores and behavioral markers of model-based choice of *r* = 0.45 (cf. Smittenaar et al., 2013) we performed a priori Monte Carlo simulations (*n* = 100.000), which showed that our sample size provides a reasonable 71% chance for finding a significant effect.

We used *R version 3.0.0* (R Development Core Team, 2013) for data analysis. Following Daw et al. (2011), we performed both analyses on the repetition probability of step 1 choices, and model-based analyses.

### Step 1 repetition probabilities

In a first step we focused on the within-subject probabilities for repeating step 1 choices. The rationale for analysing step 1 repetition probabilities is as follows (see also Figure 1). In model-free choice (habitual system), the choice probability depends on past reinforcements only. A previously rewarded step 1 choice will tend to be repeated irrespective of whether it led to reinforcement through a common or rare transition, and hence lead to a main effect of reinforcement on the previous trial (but no effect of frequency and no interaction between frequency and reinforcement). A simple single-subject measure of the model-free contribution is thus given by the main effect of reward:

P(repeat(t)|reward(t−1))−P(repeat(t)|no reward(t−1)).

Conversely, the model-based system is sensitive to the transition probabilities. Subjects who exhibit model-based strategies will prefer a switch after a rare transition is rewarded or a common transition is punished; and will tend to stay after a common transition is rewarded or a rare transition punished. Thus, a similarly simple measure of the model-based contribution is the single-subject interaction between reward and frequency.

Step 1 repetition probabilities (repetition = 1 vs. switch = 0) at trial N were predicted by *reward* (i.e., reward = +0.5 vs. no reward = −0.5) and *transition* (common = +0.5 vs. rare = −0.5) at the preceding trial (N − 1) using a baseline logistic linear mixed-effects model (GLMM; Pinheiro and Bates, 2000; fitted using the *glmer* function from the *lme4* package; Bates et al., 2013; using a maximal random effects structure; Barr et al., 2013). For statistical testing, we use unstandardized orthogonalized logistic regression coefficients, *b*, reflecting an unstandardized effect size (on a logit scale), together with 95% confidence intervals (CIs) based on posterior simulations (*n* = 10.000 per model; under a flat prior) using the *sim* function in the *arm* package (Gelman et al., 2013). To enable comparison of effect sizes between cognitive abilities, we moreover report (exponential) standardized regression coefficients β. The effects of the five cognitive ability scores on repetition-probabilities were tested by separately adding linear and quadratic terms of each ability score. For models where all effects involving a quadratic trend were non-significant, quadratic terms were removed from the model. Median- and tertile-splits of the ability scores were computed for plotting.

We tested the effects of each cognitive ability measure on individual choice strategies by computing separate generalized linear mixed-effects models (GLMM; see Table 2 for the results). We tested the effects of all five ability measures on the main effect of *reward* and the *reward* × transition interaction, and corrected *p*-values for the *False Discovery Rate* (*FDR*; Benjamini and Hochberg, 1995) for the 10 tests (5 measures × 2 linear/quadratic effects). In an explorative manner, we additionally tested the main effect of cognitive abilities on overall repetition probabilities—reflecting choice stickiness—and whether cognitive abilities moderated the *transition* effect, again correcting FDR for 10 tests.

### Computational modeling

We fitted the original computational model by Daw et al. (2011) using a mixed-effects fitting procedure (Huys et al., 2012). The model contains seven free parameters: the inverse temperature parameters at the first- (β_1_) and second-stage (β_2_) that control how deterministic choices are; the first- (α_1_) and second-stage learning rate (α_2_); the relative degree of second-stage prediction errors to update first-stage model-free values (λ); the weighting parameter (ω) determining the balance between model-free (ω = 0) and model-based (ω = 1) control; as well as *p*, which captures first-order perseveration. Table 3 displays the estimated parameter values. We then tested the effects of the cognitive ability scores on individual parameter estimates using correlations and linear models. Due to the normal distribution assumption in parameter fitting and statistical analysis we transformed bounded model parameters to an unconstrained scale via a logistic transformation [*x*′ = *log*(*x*/(1 − *x*))] for parameters α_1_, α_2_, λ, and ω and via an exponential transformation [*x*′ = exp(*x*)] for parameters β_1_ and β_2_.

## Results

Table 1 provides the summary statistics for the cognitive ability measures. There was a large range of cognitive abilities. DSST and TMTspeed scores were highly correlated, suggesting that both measures share variance related to processing speed (Corrigan and Hinkeldey, 1987; Sánchez-Cubillo et al., 2009). As expected, the executive control measure (TMTexec) was largely independent of perceptual speed and working memory (Corrigan and Hinkeldey, 1987; Arbuthnott and Frank, 2000; Sánchez-Cubillo et al., 2009), and working memory functioning exhibited only small and statistically non-significant correlations with processing speed (Li and Lewandowsky, 1995). Verbal knowledge correlated with processing speed, suggesting some amount of shared variance related to general intelligence between these measures (Johnson and Bouchard, 2005).

**Table 1:** **Summary statistics of fluid intelligence scores**.

**Task**	**DSST**	**TMTspeed**	**TMTexec**	**Digit span backwards**	**MWT-B**
*Mean (SD)*	68.6 (15.9)	34.2 (12.1)	2.2 (0.76)	7.6 (2.6)	32 (3.2)
*Range (Min − Max)*	35–98	20–70	1.1–4.7	4–14	24–37
*Correlations*					
TMT speed	−0.575^**^				
TMT exec	−0.099	−0.282			
Digit Span Backwards	0.306	−0.121	−0.090		
MWT-B	0.576^**^	−0.490^**^	−0.205	0.379^+^	

** *p < 0.01*;

+ *p < 0.1*;

*DSST, Digit Symbol Substitution Task score; TMTspeed, Trail Making Test A in s; TMTexec, Trail Making Test B in s/TMTspeed); Digit Span, Digit Span Backwards maximum span retained; MWT-B, German vocabulary test*.

In the two-step task, we found a main effect of *reward* (*b* = 0.68, 95% CI [0.42 0.94], exp(β) = 1.97, *p* < 0.001) and a *reward* × transition interaction (*b* = 1.15 [0.55, 1.72], exp(β) = 3.16, *p* < 0.001), indicating contributions of both model-free and model-based strategies to our data. The main effect of transition (*b* = 0.15 [–0.01 0.34], exp(β) = 1.16, *p* = 0.06) did not reliably differ from zero. There was substantial inter-individual variance in all three measures (1.34 ≥ *SD_b_* ≥ 0.25).

### Measures of cognitive abilities are associated with both model-based and model-free decisions

**Figure 2:**
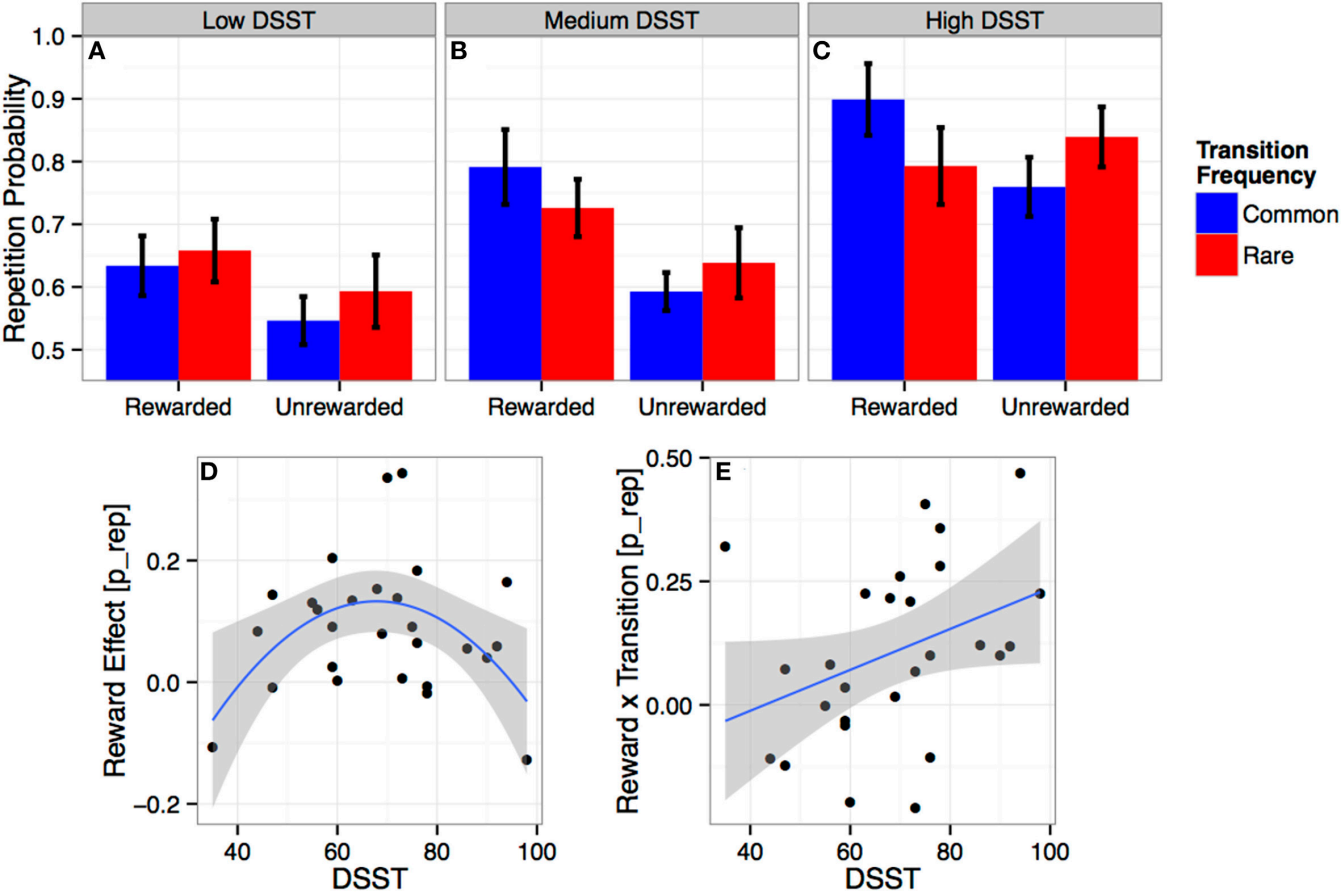
**(A–C)** Choice repetition probabilities: Average proportion of trials on which participants repeated their previous choice, as a function of outcome (reward vs. no reward) and transition (common vs. rare) at the previous trial. Results are presented for individuals with a *low* (**A**, 35–59), *medium* (**B**, 59–75), and *high* (**C**, 76–98) performance score on the *Digit Symbol Substitution Test* (*DSST*). Error bars are subject-based standard errors of the means. **(D–E)** Individual reward and transition effects and DSST performance: Individual estimates of the main effect of *reward* (= rewarded − unrewarded; **D**) and the *reward* × transition interaction (= rewarded common − rewarded rare − unrewarded common + unrewarded rare; **E**) on repetition-probabilities (*p_repeat*: repetition = 1, switch = 0) as a function of individual *DSST* scores. Lines show the estimated quadratic **(D)** and linear **(E)** effects with 95% confidence intervals.

Several aspects of cognitive abilities affected the strength of model-based decision-making as measured by the *reward* × transition effect. There was a significant three-way interaction between *linear DSST, reward*, and *transition* (*b* = 59 [21 96]; for *p*-values see Table 2), indicating more model-based choices in high speed (DSST) subjects (see Figures 2A–C,E). Similar three-way interactions involving *linear TMTspeed* (*b* = −47 [−83 −11]; see SOM Figure S1A) and *linear MWT* (*b* = 43 [5 81]; see SOM Figure S1B) also showed more model-based behavior with increasing *TMTspeed* and *MWT*, but did not survive FDR correction (*p* < 0.10, see Table 2). There was no significant effect for any other cognitive ability measure.

**Table 2:** **Logistic mixed-effects model results testing the effects of individual cognitive abilities on reward, transition frequency, and their interaction in first-stage choice repetition**.

	**DSST**			**TMTspeed**			**MWT**		
	**exp(β)**	***P*_*unc***	***P*_*FDR***	**exp(*β*)**	***P*_*unc***	***P*_*FDR***	**exp(*β*)**	***P*_*unc***	***P*_*FDR***
Main effect ability									
ability linear	1.65	0.003^**^	0.03^*^	1.43	0.06^+^	0.11	1.47	0.04^*^	0.09^+^
ability quadratic	0.81	0.02^*^	0.08^+^						
Reward × ability									
ability linear	1.18	0.096^+^	0.48	1.12	0.35	0.83	1.11	0.43	0.83
ability quadratic	0.78	0.002^**^	0.02^*^						
Transition × ability									
ability linear	1.20	0.02^*^	0.08^+^	1.12	0.17	0.42	1.07	0.44	0.63
ability quadratic	0.92	0.14	0.42						
Reward × Transition × ability									
ability linear	2.23	0.003^**^	0.03^*^	1.89	0.02^*^	0.06^+^	1.79	0.03^*^	0.10^+^
ability quadratic	–	–	–						

+ *p < 0.10*;

* *p < 0.05*;

** *p < 0.01*;

^***^p < 0.001; exp(β), exponential standardized logistic regression coefficient, indicating the (multiplicative) influence of cognitive ability scores on odds-ratios; p-values indicate significance: P_unc_, uncorrected; P_FDR_, FDR corrected; DSST, Digit Symbol Substitution Task score; TMT, Trail Making Test A in s × −1; MWT, German vocabulary test score (Mehrfachwahl-Wortschatz-Intelligenz Test). Main effect ability reflects choice stickiness. Reward indicates whether participants were rewarded or not on the preceding trial, reflecting model-free learning. Transition indicates whether previous transition was common or rare. The reward × transition interaction reflects model-based learning. BothTMTexec (= Trail Making Testexecutive) and Digit Span Backwards did not significantly interact with reward, transition, or their interaction and results from these variables are presented in the SOM. Control analyses showed that all significant effects survived statistical control for years of education as well as its interactions with reward, transition, and reward × transition.

**Table 3:** **Computational mixed-effects model parameter estimates**.

**Parameter**	**β_1_**	**β_2_**	**α_1_**	**α_2_**	**λ**	**ω**	***p***
Mean	5.00	3.63	0.39	0.25	0.45	0.49	0.12
Subject SD	0.46	0.18	0.34	0.51	0.44	0.80	0.16

*Standard Deviations (SD) of the parameters are given on the transformed scale used for parameter fitting and statistical analysis. Statistical tests for model components are based on Bayesian model comparison (see SMO). Subject SD indicates variability of estimated model parameters across individual participants. In the model fitting, we allowed all model parameters to vary across subjects; this procedure effectively de-confounds effects of cognitive abilities on model parameters from any other process captured in the computational model*.

There were also associations with model-free performance measured in terms of the main reward effect. There was a significant interaction between *reward* and *quadratic DSST* (*b* = −22 [−36 −8]; see Figure 2D), indicating that individuals with a medium level of processing speed, i.e., *DSST* performance, (Figure 2B) showed strongly model-free behavior (i.e., strong main effect *reward*), which was reduced or absent for *high-* (Figure 2C) or *low-* (Figure 2A) *DSST* participants. No other interaction between *cognitive abilities* and the effect of *reward* was significant.

Exploratively, we also tested whether cognitive abilities interact with *transition and stickiness*. There were no significant effects involving transition. Stickiness (indicated by the average repetition probability) did increase linearly with *DSST* (*b* = 38 [13 62]). Effects of other cognitive abilities did not survive correction for multiple comparisons.

### Measures of cognitive abilities modulate the tradeoff between model-based and model-free decisions

To directly examine how variation in DSST is associated with the tradeoff between model-free and model-based behavior, we first computed a difference score that measured each participant's relative preference for model-based over model-free behavior: w_repeat_ = “*reward* × transition” − “*reward*” (cf. Smittenaar et al., 2013). There was a marginal linear (*b* = 0.34 [−0.04 0.72], *p* = 0.08) but a significant quadratic (*b* = 0.49 [0.10 0.88], *p* = 0.02) association between DSST and w_repeat_, reflecting a shift from model-free control in *low-DSST* participants to model-based control in *high-DSST* participants.

The above analyses consider the effects of reward and transition on the next choice, but ignore more long-term effects. Reinforcement learning approaches (Sutton and Barto, 2009) allow explicit formulation of learning and decision-processes and thus test their ability to account for the entire dataset. We fitted such a model to the choice data. It comprised model-free and model-based components (Daw et al., 2011), and a parameter ω for the tradeoff between the two systems.

**Figure 3:**
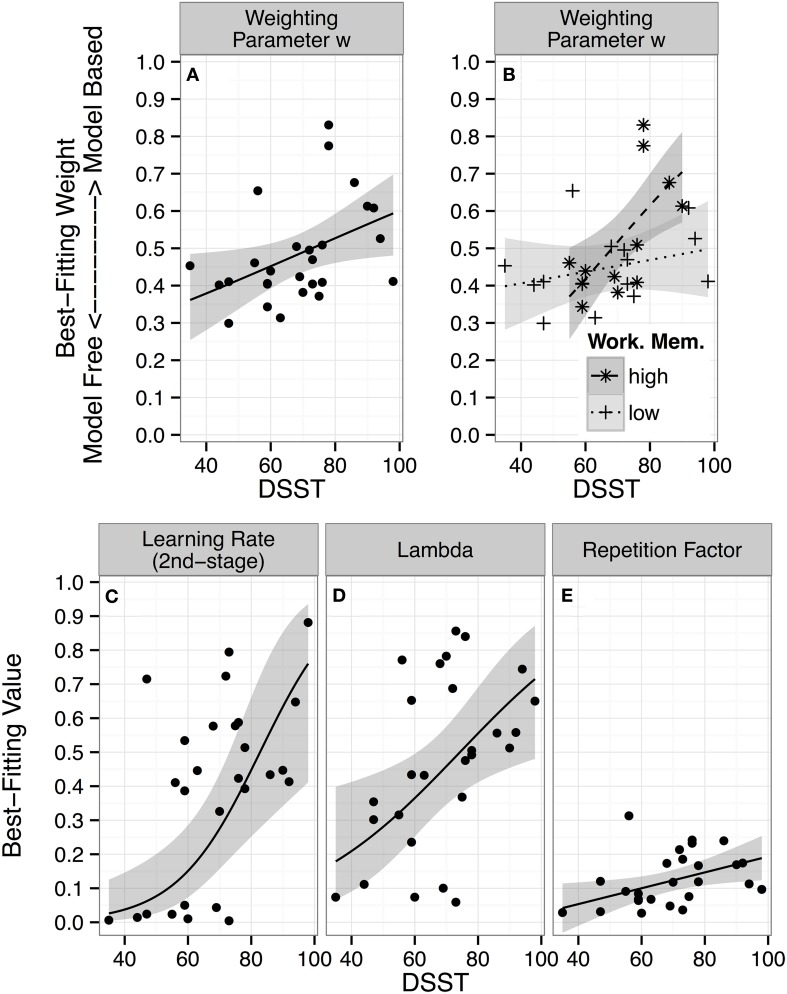
**Individual parameter estimates and DSST performance: Maximum posterior parameter values of the dual-system reinforcement learning model for each participant as a function of performance on the *Digit Symbol Substitution Test* (*DSST*) are displayed**. The lines represent predictions from linear regressions of each model parameter on DSST scores, with 95% confidence intervals (CI). **(A–D)** Regression lines and CI in unbounded fitting-space were transformed to model-space for plotting by passing them through the inverse-logit function. **(A)** Best-fitting individual parameter values for the weighting parameter ω, which determines the balance between model-free (weight = 0) and model-based (weight = 1) control. **(B)** Regression of best-fitting weighting parameter values on the interaction between DSST scores × working memory span (median-split factor). **(C)** Best-fitting parameter values for the second-stage learning rate α_2_. **(D)** The lambda (λ) parameter determines update of model-free step 1 action values by step 2 prediction errors. **(E)** Repetition factor, *p*, indicates how strongly individuals tend to repeat previous actions.

There was a linear correlation between DSST and individual ω parameter estimates (*r*_(25)_ = 0.42 [0.04 0.68], *p* = 0.03; see Figure 3A), confirming that *high-DSST* participants relied more on model-based and *low-DSST* more on model-free learning.

There was no main effect of working memory functioning. However, given recent accounts whereby the balance between model-free and model-based control is moderated by working memory (Otto et al., 2013b; Smittenaar et al., 2013), we asked whether working memory functioning might moderate the DSST effect. We split the Digit Span Backwards score along its median, and regressed individual ω estimates on the two-way interaction of the continuous DSST score with the categorical Digit Span factor (high vs. low). This revealed a significant *DSST* × *Digit Span Backwards* interaction (*b* = 2.73 [0.18 5.26], β = 0.53, *p* = 0.03, *R*^2^ = 0.36; for the continuous interaction: *p* = 0.12). Figure 3B shows that large values of ω are achieved only when high DSST scores are paired with a high working memory functioning (DSST effect: *b* = 3.24 [1.03 5.46], β = 0.64, *p* = 0.006), whereas high DSST performance does not enhance model-based relative to model-free control for individuals with low working memory functioning (DSST effect: *b* = 0.51 [−0.67 1.71], β = 0.10, *p* = 0.37).

The effects of *DSST* for individuals with a high working memory functioning (*b* = 3.09 [0.30 5.86], β = 0.61, *p* = 0.03) and the *DSST* × *Digit Span Backwards* interaction (*b* = 2.83 [0.02 5.69], β = 0.56, *p* < 0.05) on ω remained significant when controlling for other cognitive ability scores, suggesting that the effects were unique to the two scores and independent from the other abilities.

Finally, we explored whether DSST was associated with any other model parameter and FDR corrected *p*-values for the six tests. There were three significant correlations: First, with the second stage learning parameter α_2_ (*r*_(25)_ = 0.57 [0.24 0.78]; *p* = 0.002 uncorrected; *p* = 0.01 corrected; Figure 3C), reflecting faster second stage learning in high- compared to low-DSST participants; second, with the λ parameter (*r*_(25)_ = 0.47 [0.11 0.72]; *p* = 0.01 uncorrected; *p* = 0.04 corrected; see Figure 3D), indicating stronger update of model-free step 1 action values by step 2 prediction errors in high- compared to low-DSST participants; and third, with the stickiness parameter *p*(*r*_(25)_ = 0.44 [0.07 0.70]; *p* = 0.02 uncorrected; *p* = 0.04 corrected; Figure 3E), reflecting predisposition to stronger choice stickiness.

### Overall performance

Model-based decisions are more effective in the two-step task. Indeed, subjects with higher DSST scores had achieved more rewarded trials (*r*_(25)_ = 0.38 [0.01 0.67], *p* < 0.05; see SOM Figure S2). This effect disappeared when controlling for the weighting parameter (ω; partial correlation: *r*_(25)_ = 0.27 [−0.12 0.59], *p* = 0.17; correlation between ω and rewarded trials: *r*_(25)_ = 0.38 [−0.004 0.66], *p* = 0.053), indicating that high-DSST subjects increased their reward by relying on model-based control.

## Discussion

We examined how cognitive abilities were related to model-free and model-based components of decision-making using a task specifically designed to dissociate these components (Daw et al., 2011). We found that specific abilities are differentially related to model-based and model-free choice.

The central finding is that processing speed considerably enhanced model-based over model-free choice behavior. Participants with higher DSST and TMT speed scores had higher markers of model-based behavior (c.f. Arbuthnott and Frank, 2000; Joy et al., 2003). This finding is in line with theoretical accounts of model-based and model-free decision-making, which propose that model-based predictions are computationally expensive and time consuming (Daw et al., 2005; Keramati et al., 2011; Huys et al., 2012). To determine the value of an action, the model-based system considers each possible outcome for this action: it computes how likely each outcome is, what its expected value will be, and then integrates this information to estimate overall action value (see SOM, Equation 4). Model-free action values, in contrast, are pre-computed, stored in memory and readily available for choice.

There are several prominent accounts of the arbitration between the two systems. Daw et al. (2005) suggested that arbitration depends on the relative certainty of the predictions made by the two systems. Cognitive abilities could affect this in a number of ways. First, considering delay discounting, Kurth-Nelson et al. (2012) suggested that cognitive abilities could influence the search process. Under time pressure, lower processing speed might lead to poorer model-based predictions due to incomplete calculations. Second, Keramati et al. (2011) suggested that arbitration is determined by the value of information and reflects tradeoffs between speed and accuracy. Lower processing speed might make it more expensive to perform model-based evaluations. It might be possible to disentangle these possibilities by systematically exploring the effect of time pressure and uncertainty in the two systems (Lee et al., 2014). Third, system damage could also impair the functioning of either system and lead to a bias away from it (Redish et al., 2008), though this is unlikely to be relevant to the current sample of healthy subjects. Finally, arbitration between systems may be instantiated through an external system (Rich and Shapiro, 2009; Lee et al., 2014), or may be guided by self-consistency of each system's action proposal (Van der Meer et al., 2012).

Of note, the actual cognitive computations probed by the DSST are somewhat similar to those probed by model-based choices in the present task. Performing the DSST efficiently requires associating complex shapes with numbers, while in the two-step task, choices at the first step can in part be driven by the association of action sequences with numbers (the reward outcomes; Dezfouli and Balleine, 2012, 2013; Huys et al., 2014). That is, model-based control might relate to individual differences in the ability to manipulate complex sequence information, and hence reduce the number of computations needed to compute model-based predictions. Indeed, this link has been proposed previously with respect to general cognitive abilities, whereby a key aspect of intelligence is the ability to subdivide complex tasks into larger chunks (Bhandari and Duncan, 2014). The correlation between DSST and the parameter λ in the model (which mediates the effect of rewards on first-stage choices) may be seen in this light, and suggests that high processing speed may help to subdivide the complex two-step task into an efficient cognitive task representation, which closely links first-stage actions to their associated reward outcomes at terminal states.

The findings with respect to DSST are qualified by the findings involving working memory functioning. The specific task used in our study reflects both working memory capacity and manipulation of information within working memory (Li and Lewandowsky, 1995). Beyond the effects of processing speed, we did not find a strong effect of working memory functioning *per se*. We did however find that processing speed and working memory functioning interacted to moderate the tradeoff between model-based and model-free choices. Only people with high working memory functioning benefited from processing speed advantages to reach high values of model-based control. This suggests that the ability to compute model-based predictions (i.e., planning) and to manipulate complex task chunks depends on a substantial working memory functioning, and implies that dual-task manipulations (Otto et al., 2013a) may interfere with this prerequisite working memory capacity and the ability to manipulate complex representations. Furthermore, the complex spatial nature of the two-step task may require the ability to manipulate information within visuospatial working memory, which has been related to performance on backward digit span as used in our study (Li and Lewandowsky, 1995). More generally, it indicates that working memory functioning in that context represents a necessary but not a sufficient prerequisite for model-based choice behavior.

Interestingly, we also found a relation of the knowledge-based aspects of intelligence (as evinced by the vocabulary test) with model-based control. Knowledge-based or crystallized intelligence reflects accumulated knowledge about the world, i.e., it provides the kinds of world models that model-based performance may rely on. On the other hand, high verbal knowledge might support verbal task-coding in the model-based system, reflecting either spontaneous construction of verbal strategies or improved comprehension of instructions, i.e., by promoting model-construction based on verbally transmitted information.

With respect to model-free choice behavior, we found evidence for a quadratic relationship between processing speed and model-free choice. We here speculate that this quadratic effect may relate to two distinct influences of processing speed on (*i*) successful learning of the space of states and actions in the task environment and on (*ii*) relative reliance on the model-free (as compared to the model-based) system. Model-free learning algorithms depend on correct representations of the states and actions in the current environment, which needs to be learned from rather complex sequences of events in the two-step task. Here, a minimum level of processing speed may be needed for this learning to succeed and to support successful model-free learning. Higher levels of processing speed, to the contrary, may reduce the influence of model-free values on choice and induce a shift toward model-based behavior.

Interestingly, the pattern of results does not map onto either conceptualization of intelligence in any simple manner. In terms of the two-component model (Horn and Cattell, 1966; Li et al., 2004; Sternberg, 2012), we found evidence for an association between measures of fluid intelligence and model-based choice behavior, but we also found a trend association with crystallized intelligence. Likewise, for the verbal-perceptual-image model of intelligence (Johnson and Bouchard, 2005), we found associations between all components and model-based choice.

## Conclusions

In conclusion, specific aspects of individual variation in cognitive abilities are associated with individual variation in model-free vs. model-based decision-making and point toward potentially important components of the reinforcement learning process. This work also provides a bridge between procedurally defined cognitive ability components and neurocomputationally defined ones as in the two-step task, and paves the way for more detailed process-oriented models of neurocomputationally well-defined decisions. As the tradeoff between model-based and model-free decision-making is of great interest in the investigation of a variety of disorders (Everitt and Robbins, 2005; Gillan et al., 2011; Sebold et al., 2014; Sjoerds et al., 2014), this study highlights the importance of detailed measures of cognitive abilities, which may provide potential moderating or protective factors for aberrant decision-making processes associated with neuropsychiatric diseases.

### Conflict of interest statement

The authors declare that the research was conducted in the absence of any commercial or financial relationships that could be construed as a potential conflict of interest.
