# Supplementary material for: Processing speed enhances model-based over model-free reinforcement learning in the presence of high working memory functioning
Source: Front Psychol. 2014 Dec 17;5:1450. doi: 10.3389/fpsyg.2014.01450 (PMC4269125; doi:10.3389/fpsyg.2014.01450)
Supplement: Supplementary file 1 [file Presentation1.PDF]

SUPPLEMENTARY INFORMATION

SCHAD, JÜNGER, SEBOLD, GARBUSOW, BERNHARDT, JAVADI, ZIMMERMANN, SMOLKA, HEINZ, RAPP & HUYS

Title: Processing speed enhances model-based over model-free reinforcement learning in the presence of high working memory functioning

1. Results for TMT speed and MWT

Figure S1 shows results on repetition probabilities for the *TMT speed* and the *MWT*, which did not survive correction for multiple comparisons.

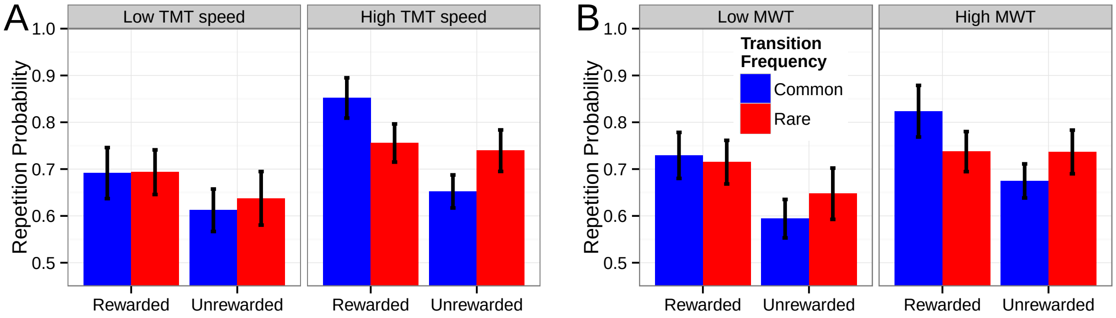

Figure S1. Average proportion of choice repetition on the current trial as a function of *Reward* (reward vs. no reward) and *Transition* (common vs. rare) on the previous trial. *A*. Results for individuals with a low (left panel) versus high (right panel) speed in the trail making test (*TMTspeed*; split at the median score of 33). *B*. Results for individuals with a low (left panel) or high (right panel) German vocabulary test score (*MWT*; split at the median score of 104).

2. Results for overall reward

We found a linear increase for the percentage of rewarded trials with higher DSST scores (see Figure S2).

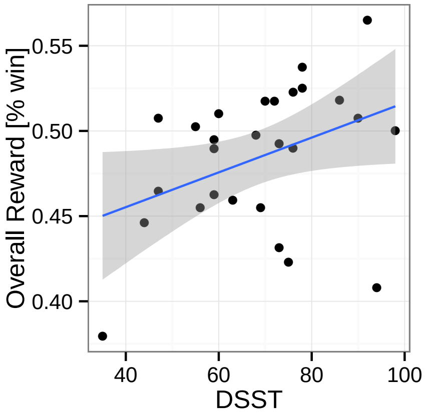

FIGURE S2. Percentage of rewarded trials per participant as a function of DSST. The line displays predictions from a linear regression (grey area = S.E.M).

3. Computational Model

We denote the state x visited in trial t as $s_{x,t}$, with state index x denoting states at first stage, $x = 1$, at second stage, $x = \{21,\ 22\}$, and final outcome, $x = \{31\ ..\ 34\}$. Actions $a_{x,t} = \{1, 2\}$ lead to probabilistic ($a_{1,t}$) or deterministic ($a_{2,t}$) transitions between successive states. Final states $s_{3,t}$ are associated with probabilistic rewards r_t, while $r_t = 0$ for all non-final states.

To account for habitual learning we used the model-free reinforcement learning algorithm SARSA(λ) temporal difference learning (Rummery & Niranjan, 1994), where action values $Q_{Hab}(s_{x,t}; a_{x,t})$ are updated based on the reward prediction error (RPE),

$$\delta_{x,t} = Q_{Hab}(s_{x+1,t}; a_{x+1,t}) + r_{x,t} - Q_{Hab}(s_{x,t}; a_{x,t}) \tag{1}$$

via

$$Q_{Hab}(s_{x,t}; a_{x,t}) = Q_{Hab}(s_{x,t}; a_{x,t}) + \alpha_x \delta_{x,t}\ , \tag{2}$$

with free learning parameters α_x. Based on analyses by Daw, Gershman, Seymour, Dayan, and Dolan (2011) we used separate learning rates α_1 and α_2 for 1st- and 2nd- stages. We allowed reward-feedback to travel across stages in the model-free system such that RPEs at the final state are used to additionally update 1st-stage action values via the free eligibility parameter λ (Sutton & Barto, 1998):

$$Q_{Hab}(s_{1,t}; a_{1,t}) = Q_{Hab}(s_{1,t}; a_{1,t}) + \alpha_1 \lambda \delta_{2,t}\ . \tag{3}$$

The habitual system thus does not explicitly represent the structure of the state environment, but instead updates reward predictions in a model-free manner.

The goal-directed system, to the contrary, constructs a model of the states, actions and transitions between states to re-compute 1st-stage action values $Q_{GD}(s_{1,t};a_{1,t})$ at each trial by weighting each actions' expected outcomes by the probability of their occurrence:

(4)
$$Q_{GD}(s_{1,t};a_{1,t}) = P(s_{21,t} \mid s_{1,t};a_{1,t}) \max_a Q(s_{21,t};a_{21,t}) + P(s_{22,t} \mid s_{1,t};a_{1,t}) \max_a Q(s_{22,t};a_{21,t}).$$

Before the experiment, participants were instructed that transition probabilities given 1st-stage action $a_{1,t} = 1$ would be 0.3 and 0.7 for the transitions to 2nd stage states $s_{21,t}$ and $s_{22,t}$ (and vice versa for $a_{1,t} = 2$). We therefore modelled the acquisition of transition probabilities by assuming participants learned to map action-state pairs $\{a_1, s_{2x}\}$ to transition probabilities, $p = \{0.3, 0.7\}$, by counting whether they had more often encountered transitions $\{a_1 = 1, s_{21}\}$ and $\{a_1 = 2, s_{22}\}$ or transitions $\{a_1 = 1, s_{22}\}$ and $\{a_1 = 2, s_{21}\}$ and concluding the more frequent category corresponds to $p = 0.7$. We assume that model-free and model-based action values are integrated at 1st-stage via

$$Q_{Int}(s_{1,t};a_{1,t}) = (1-\omega)Q_{Hab}(s_{1,t};a_{1,t}) + \omega Q_{GD}(s_{1,t};a_{1,t}) \ , \tag{5}$$

where the free weighting parameter ω determines the relative contribution of model-free and model-based action values, with a parameter value of $\omega = 0$ reflecting pure model-free control and $\omega = 1$ reflecting pure model-based control. Action values at 2nd-stage do not differ between systems and $Q_{Int}(s_{2,t};a_{2,t}) = Q_{Hab}(s_{2,t};a_{2,t}) = Q_{GD}(s_{2,t};a_{2,t})$.

Integrated action values are then used to determine action probability via the softmax function

$$P(a_{x,t} = a \mid s_{x,t}) = \frac{\exp(\beta_x[Q_{Int}(s_{x,t};a_{x,t}) + \rho \ repe(a)])}{\sum_A \exp(\beta_x[Q_{Int}(s_{x,t};A_{x,t}) + \rho \ repe(a)])} \ . \tag{6}$$

The free parameters β_x determine how deterministic choices are and capture noise and exploration. For a parameter value of $\beta_x = 0$ choice behaviour is fully random; for a value of $\beta_x = \inf$ choice is fully deterministic in the sense that higher-valued options are always preferred, even if the value-difference between options is negligible; a value of $\beta_x = 1$ reflects probability/value matching. Indicator variable repe(a) codes whether the current action $a_{1,t}$ has been chosen on the last trial [for $a_{1,t} = a_{1,t-1} \rightarrow repe(a) = 1$ and for $a_{1,t} \neq a_{1,t-1} \rightarrow repe(a) = 0$]. The free parameter ρ thus captures perseveration or stickiness of 1st-stage choices, and positive values of ρ reflect an enhanced probability of repeating a 1st-stage action on the consecutive trial.

The model overall contains seven free parameters $\underline{\theta} = (\beta_1, \beta_2, \alpha_1, \alpha_2, \lambda, \omega, \rho)$, and corresponds to the one introduced by Daw et al. (2011).

4. Mixed effects model fitting

We used Bayesian modeling to estimate the model parameters θ_i for individual participants i (cf. Huys et al., 2011). Combining the likelihood with information from a prior regularizes and stabilizes estimates particularly when the estimates are not well constrained by the data.

We estimated the maximum a posteriori (MAP) parameters for each participant via

$$\hat{\theta}_i^{MAP} = \underset{\theta}{\operatorname{argmax}}\ P(A_i \mid \theta_i) P(\theta_i \mid \mu_\theta, \sigma_\theta)\ , \tag{7}$$

where A_i are all the actions by subject i and the parameters μ_θ and σ_θ are the mean and the variance of the prior Gaussian distribution. We assume the likelihood $P(A_i \mid \theta_i) = \prod_{t=1:nt} P(A_{i,t} \mid \theta_i)$, reflecting independence of individual actions given the model and the parameters.

We moreover used empirical Bayes and set the prior parameters μ_θ and σ_θ to the maximum likelihood estimates for all N subjects:

$$\begin{aligned} \hat{\mu}_\theta^{ML}, \hat{\sigma}_\theta^{ML} &= \underset{\mu,\sigma}{\operatorname{argmax}}\ P(A \mid \mu_\theta, \sigma_\theta) \\ &= \underset{\mu,\sigma}{\operatorname{argmax}} \prod_{i=1}^{N} \int d\theta_i P(A_i \mid \theta_i) P(\theta_i \mid \mu_\theta, \sigma_\theta)\ , \end{aligned} \tag{8}$$

where $A = \{A_i\}_{i=1}^N$.

We find the ML estimates via an EM algorithm. In the E-step, we perform a Laplace approximation to the individual posterior distributions of model parameters:

$$P(\theta_i \mid A_i) \approx N(\theta_i^{MAP(k)}, \theta_i^{\sigma(k)})\ , \tag{9}$$

where $N(\theta_i^{MAP(k)}, \theta_i^{\sigma(k)})$ denotes a normal distribution over θ_i at each step k of the EM algorithm, with mean $\theta_i^{MAP(k)}$ and approximate variance $\theta_i^{\sigma(k)}$, which is derived from the Hessian of the posterior at its maximum $\theta_i^{MAP(k)}$.

In the M-step, we use this approximation to optimize for μ_θ and σ_θ: differentiating the likelihood (Equation 8) with respect to μ_θ and σ_θ yields updates for the prior mean and variance as

$$\mu_\theta^{(k)} = \frac{1}{N} \sum_i \theta_i^{MAP(k)} \tag{10}$$

$$\sigma_\theta^{(k)} = \frac{1}{N} \sum_i [(\theta_i^{MAP(k)})^2 + \theta_i^{\sigma(k)}](\mu_\theta^{(k)})^2. \tag{11}$$

Validity of these fitting procedures was confirmed in Monte Carlo simulations using simulated data with a known decision process.

5. Model validation

We asked whether the computational dual-control model, which assumes contributions of separate model-free and model-based systems to behavioral choice, was supported for our present data. To this end, we fitted six different decision models to the data assuming

different combinations of a model-based system, a model-free system, and a tendency to repeat previous first-stage actions (for the list of models see Figure S3). We wanted to determine which model was most supported by the data, where good models are able to explain the current data without being able to explain very different data as well (cf. Huys et al., 2011).

Ideal Bayesian model comparison relies on the posterior log probability $\log P(M \mid A)$ of each model M given the observed choices A. We assumed a flat prior on the models, reflecting the assumption that all models were equally likely a priori. For model comparison we therefore instead used the model likelihood $\log P(A \mid M)$ of the data given each of the models. This likelihood involves integrals over parameters at the prior group and at the individual subject level. We approximated the group-level integral via (Kass & Raftery, 1995):

$$\begin{aligned} \log P(A \mid M) &= \int d\mu_\theta \int d\sigma_\theta \, P(A \mid \mu_\theta, \sigma_\theta) P(\mu_\theta, \sigma_\theta \mid M) \\ &\approx -\frac{1}{2}\mathrm{BIC}_{\mathrm{int}} = \log P(A \mid \hat{\mu}_\theta^{ML}, \hat{\sigma}_\theta^{ML}) - \frac{1}{2}\,|M| \log(|A|) \end{aligned} \tag{12}$$

The log likelihood $\log P(A \mid \hat{\mu}_\theta^{ML}, \hat{\sigma}_\theta^{ML})$ contains integrals at the second level, i.e., over individual subject's parameters θ_i, which we approximated via importance sampling (MacKay, 2003):

$$\begin{aligned} \log P(A \mid \hat{\mu}_\theta^{ML}, \hat{\sigma}_\theta^{ML}) &= \sum_i \log \int d\mathbf{h} P(A_i \mid \mathbf{h}) P(\mathbf{h} \mid \hat{\mu}_\theta^{ML}, \hat{\sigma}_\theta^{ML}) \\ &\approx \sum_i \log \frac{1}{K} \sum_{k=1}^{K} P(A_i \mid \mathbf{h}^k) \ , \end{aligned} \tag{13}$$

where $K = 1000$ indicates the number of samples drawn from the empirical prior distribution $\mathbf{h}^k \sim P(\mathbf{h} \mid \hat{\mu}_\theta^{ML}, \hat{\sigma}_\theta^{ML})$.

Figure S3 shows the BIC_{int} scores for each of the tested models. The analysis clearly favors the full model, which includes model-free and model-based systems as well as a repetition factor, over all simpler nested models, and thus supports the dual-control model for the present data.

We computed the total "predictive probability" as

$$P(A \mid \{\hat{\theta}_i^{MAP}\}_{i=1}^{N}) = \prod_{i=1}^{N} \prod_{t=1}^{T} P(a_t^i \mid s_t, \hat{\theta}_i^{MAP}) \ . \tag{14}$$

Note that we term this probability "predictive" because it determines the probability of the next choice a_t given all previous choices by the subject. However, model predictions rely on parameters that were fitted to all individual subject's choices. We tested whether individual subjects' predictive probabilities $\sqrt[T]{P(A \mid \hat{\theta}_i^{MAP})}$ were significantly higher than chance using a binomial test. The winning model predicted choices better than chance

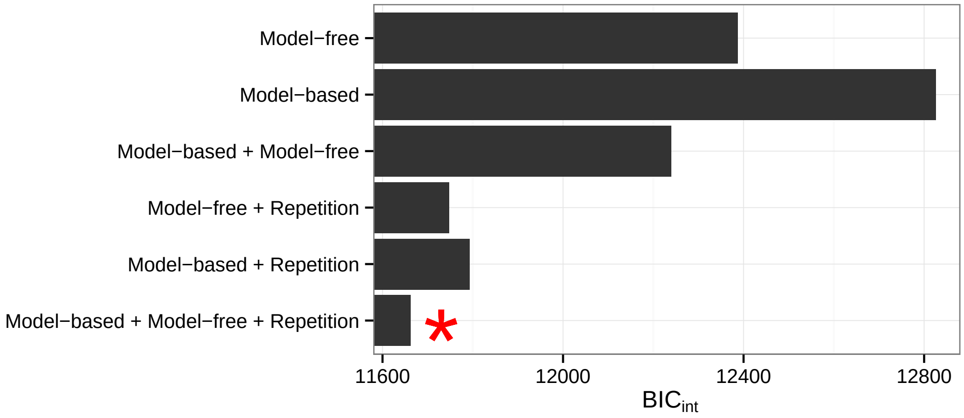

FIGURE S3. BIC_{int} scores for six models testing the presence of the three components (1) model-based control (2) model-free control and (3) a repetition factor ρ. The results clearly support the model incorporating all three components (indicated with a red star).

for 19 out of 27 subjects ($p < .10$), but did not significantly fit the data from the remaining 8 subjects ($p > .10$). A logistic regression indicated that DSST scores did not have a significant influence on whether an individual subject's model fit was successful ($b = 0.061, SE = 0.033, t = 1.85, p = .065$). We also computed the overall predictive probability for all choices by all subjects as $\sqrt[TN]{P(A \mid \{\hat{\theta}_i^{MAP}\}_{i=1}^{N})} = 0.60$, and this absolute measure of model fit highly significantly exceeded chance level ($p < .001$).

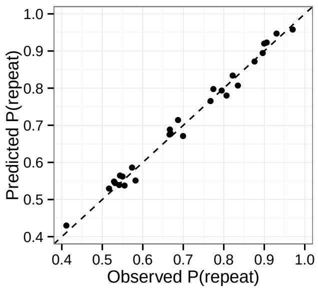

FIGURE S4. Predicted repetition probabilities as a function of observed repetition probabilities for each individual subject.

Next, we asked whether the softmax was an adequate link function for computing choice probabilities. It is visible in Figure S4 that predicted repetition probabilities closely matched observed repetition probabilities, supporting the softmax as a reasonable link function.

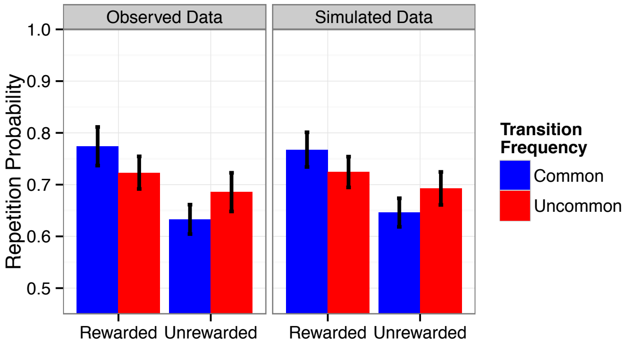

FIGURE S5. Observed (left panel) and simulated (right panel) repetition probabilities after rewarded versus unrewarded trials and common (blue) versus uncommon (red) transitions. Values were averaged across all 27 subjects, and across 100 simulation runs for each subject. The model well captures the observed pattern of a main effect of *reward* and a *reward x transition* interaction.

We further tested the winning model and the best-fitting individual subject parameters θ_i^{MAP} by using them to simulate choice data on the present two-step task. Figure S5 shows that model predictions were well in line with the observed pattern of overall results. We split the model predictions by whether the θ_i^{MAP} parameters were taken from individuals with a low, medium, or high DSST score. It is visible in Figure S6 that the model successfully captured the observed pattern of results for the DSST groups.

Overall, these results support the validity of the present modeling analyses.

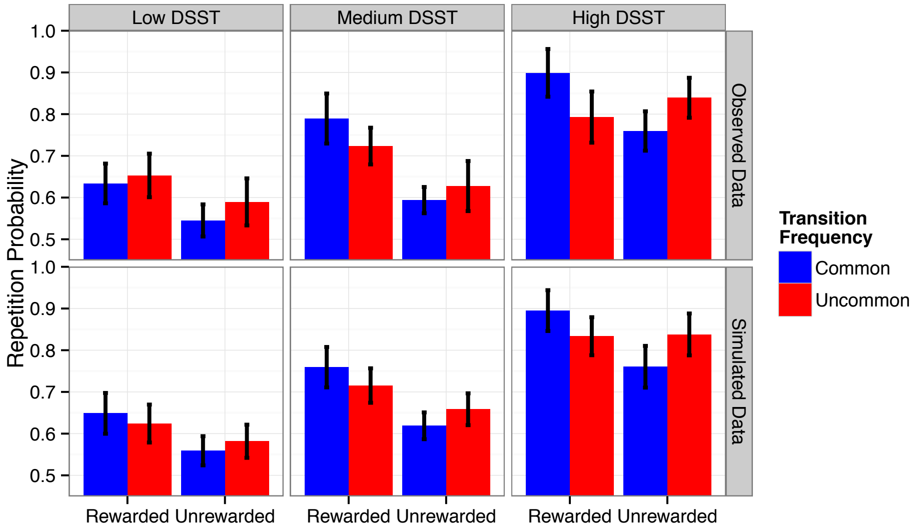

FIGURE S6. Observed (upper panels; cf. Figure 2A-C) and simulated (lower panels) repetition probabilities after rewarded versus unrewarded trials and common (blue) versus uncommon (red) transitions for three different levels of low (left panels), medium (middle panels), and high (right panels) DSST scores. Each panel contains data from $N = 9$ subjects; simulations as in Figure S5.

References

Army Individual Test Battery. (1944). *Manual of directions and scoring.* Washington, DC: War Department, Adjutant General's Office.

Daw, N. D., Gershman, S. J., Seymour, B., Dayan, P., & Dolan, R. J. (2011). Model-based influences on humans' choices and striatal prediction errors. , *69*(6), 1204-1215.

Huys, Q. J., Cools, R., Gölzer, M., Friedel, E., Heinz, A., Dolan, R. J., et al. (2011). Disentangling the roles of approach, activation and valence in instrumental and pavlovian responding. *PLoS Computational Biology*, *7*(4), e1002028.

Kass, R. E., & Raftery, A. E. (1995, 2014/02/15). Bayes factors. , *90*(430), 773-795.

Lehrl, S. (2005). *Mehrfachwahl-Wortschatz-Intelligenztest MWT-B (5th ed.).* Balingen: Spitta.

MacKay, D. J. (2003). *Information theory, inference and learning algorithms.* Cambridge University Press.

Pinheiro, J., & Bates, D. (2000). *Mixed-effects models in S and S-PLUS.* New York: Springer.

Rummery, G. A., & Niranjan, M. (1994). *On-line Q-learning using connectionist systems.* University of Cambridge, Department of Engineering.

Sutton, R. S., & Barto, A. G. (1998). *Introduction to reinforcement learning.* MIT Press.

Wechsler, D. (1997). *WAIS-III: Wechsler adult intelligence scale: Administration and scoring manual.* Psychological Corporation.
